# Modulation of HO-1 by Ferulic Acid Attenuates Adipocyte Differentiation in 3T3-L1 Cells

**DOI:** 10.3390/molecules22050745

**Published:** 2017-05-05

**Authors:** Eun-Jeong Koh, Kui-Jin Kim, Young-Jin Seo, Jia Choi, Boo-Yong Lee

**Affiliations:** Department of Food Science and Biotechnology, College of Life Science, CHA University, Seongnam, Kyeonggi 13488, Korea; kej763@naver.com (E.-J.K.); Kuijin.Kim@gmail.com (K.-J.K.); yj2477@hanmail.net (Y.-J.S.); wldk3176@gmail.com (J.C.)

**Keywords:** Ferulic acid, 3T3-L1, HO-1, adipogenesis, obesity

## Abstract

Ferulic acid (FA) is phenolic compound found in fruits. Many studies have reported that FA has diverse therapeutic effects against metabolic diseases. However, the mechanism by which FA modulates adipogenesis via the expression of heme oxygenase-1 (HO-1) implicated in suppression of adipocyte differentiation is not fully understood. We investigated whether HO-1 can be activated by FA and suppress adipogenic factors in 3T3-L1. Our results showed that FA suppresses triglyceride-synthesizing enzymes, fatty acid synthase (FASN) and acetyl-CoA carboxylase (ACC). We observed that the expression of CCAAT/enhancer binding protein α (C/EBPα) and peroxisome proliferator-activated receptor γ (PPARγ) were suppressed by FA. In addition, HO-1 inhibitor stimulated lipid accumulation, while FA attenuated lipid accumulation in 3T3-L1 treated with HO-1 inhibitor. We also observed that the expression of HO-1 had the same tendency as C/EBP homologous protein 10 (CHOP10) during the mitotic clonal expansion (MCE) of adipogenesis. We next employed siRNA against HO-1 to clarify whether HO-1 regulates CHOP10. The results indicated that CHOP10 is downstream of HO-1. Furthermore, FA-mediated HO-1/CHOP10 axis activation prevented the initiation of MCE. Therefore, we demonstrated that FA is a positive regulator of HO-1 in 3T3-L1, and may be an effective bioactive compound to reduce adipocyte tissue mass.

## 1. Introduction

Obesity is one of the major risk factors for chronic diseases, including cardiovascular disease, diabetes, and insulin resistance [1]. Similarly, increasing evidence suggests that obesity causes chronic inflammation via adipocyte-secreted paracrine factors, which prolongs physical pain and affects quality of life [2,3]. Although obesity could be treated with medical approaches and diet management, it remains a public health concern.

When there is an imbalance between food intake and energy expenditure, adipocytes store energy as intracellular lipids. This process results in enhanced adipogenesis, and consequently, obesity [4]. Recently, adipocyte differentiation is stimulated by reactive oxygen species (ROS) in 3T3-L1 cells. CCAAT/enhancer binding protein α (C/EBPα) and peroxisome proliferator-activated receptor γ (PPARγ)—adipogenic transcriptional factors—are up-regulated by ROS during adipogenesis [5,6]. The generation of ROS is modulated by anti-oxidant enzymes. Heme oxygenase-1 (HO-1) is one of the antioxidant enzymes implicated in adipocyte differentiation, and is the rate-limiting enzyme that degrades heme to biliverdin, iron, and carbon monoxide in the catabolism of heme [7,8]. In recent studies, obesity decreased the activity of HO-1, leading to an increase in the intracellular heme and adipocyte-derived inflammatory cytokines in vivo [9,10].

Several signalling pathways are involved in modulating adipocyte differentiation [11]. CCAAT/enhancer binding protein β (C/EBPβ) is one of the important transcription factors activated in the early phase of adipocyte differentiation. C/EBPβ is activated by numerous mechanisms, involving transcriptional regulation and phosphorylation during adipogenesis [12]. In particular, the dominant negative expression of C/EBP homologous protein (CHOP10) by cell cycle arrest transiently stimulates the activation of C/EBPβ undergoing terminal differentiation of adipogenesis. C/EBPα, PPARγ, and adipocyte protein 2 (aP2) are primarily required for terminal differentiation of adipogenesis [13]. Fatty acid synthase (FASN) and acetyl-CoA carboxylase (ACC) are also involved in promoting intracellular lipid synthesis in the late stage of adipogenesis [14,15]. Thus, the regulation of oxidative stress-associated enzymes and adipogenic transcription factors is important to properly attenuate the development of adipocytes in obesity.

Ferulic acid (FA) is phenolic phytochemical found in fruits, as well as in both the seeds and cell walls of commelinid plants such as rice and oats [16]. FA has various effects against metabolic diseases [17]. Recently, a study reported that FA induces the expression of HO-1 via the activation of extracellular signal-regulated kinases (ERK) and nuclear factor (erythroid-derived 2)-like 2 (Nrf2) in AHH-1 cells [18]. In addition, another group showed that FA inhibits intracellular lipid accumulation in vitro and prevents high fat diet-induced obesity in vivo [19,20]. However, the effects of FA on adipocyte differentiation are still unclear. The aim of this study was to investigate whether HO-1 can be activated by FA in adipocytes and suppress crucial adipocyte differentiation makers in 3T3-L1 cells.

## 2. Results

### 2.1. FA Suppresses Adipocyte Differentiation Factors and Intracellular Lipid Accumulation in 3T3-L1

**Figure 1:**
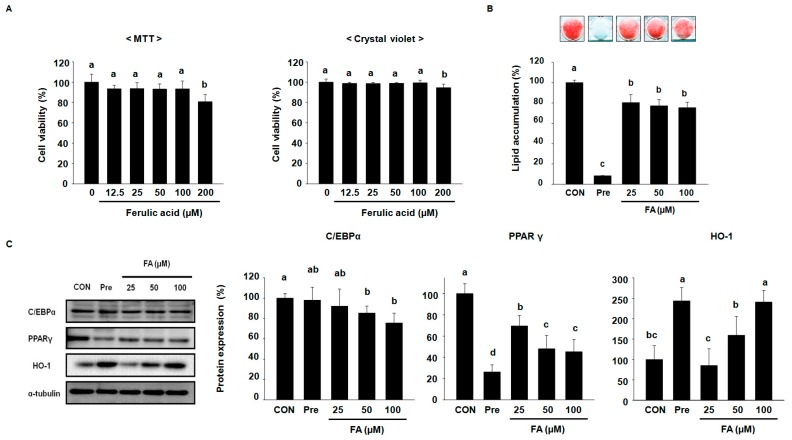
Ferulic acid (FA) suppresses adipocyte differentiation factors and intracellular lipid accumulation in 3T3-L1 cells. (**A**) 3T3-L1 pre-adipocytes were treated with up to 200 μM FA for 24 h. Effects of FA on cytotoxicity in 3T3-L1 pre-adipocytes were measured by MTT and crystal violet assay. The experiments were performed in hexa-plicate; (**B**) Lipid accumulation was measured by an Oil red O (ORO) assay in 3T3-L1 cells differentiated in the presence or absence of 25, 50, and 100 μM FA for 8 days and determined at 490 nm. The experiments were performed in hexa-plicate; (**C**) Cell lysates differentiated over 5 days were subjected to Western blots to analyze C/EBPα, PPARγ, and HO-1. The protein expression level was normalized against α-tubulin.: Results were analyzed by one-way ANOVA and Duncan′s multiple range test. The experiments were performed in triplicate. The *p*-value in the multiple comparison results (e.g., a, b, c, d, and e) indicate significant differences among the groups (*p* < 0.05).

We performed MTT and crystal violet assay to analyze the toxicity of FA on 3T3-L1 pre-adipocytes. As shown in Figure 1A, 200 μM FA was found to be toxic to cells. Therefore, the concentrations of 25, 50, and 100 μM FA were selected for further investigation. 3T3-L1 pre-adipocytes have been used to evaluate the effects of FA on adipogenesis in vitro. 3T3-L1 pre-adipocytes were stimulated with 3-isobutyl-1-methylxanthine, dexamethasone, and insulin (MDI) in the presence or absence of FA for 8 days. Oil red O (ORO) staining was performed to evaluate the effect of FA on the intracellular lipid accumulation of adipocytes. As shown in Figure 1B, ORO staining revealed that FA significantly decreased intracellular lipid accumulation compared with the corresponding control. 

The protein expression of C/EBPα and PPARγ were increased during adipogenesis of 3T3-L1 cells [11], while the expression of HO-1 were decreased. To examine the effects of FA on adipocyte differentiation, 3T3-L1 pre-adipocytes were stimulated with MDI in the presence or absence of FA. We measured the expression of C/EBPα, PPARγ, and HO-1. As shown in Figure 1C, Western blot analysis revealed that the expression of C/EBPα and PPARγ were induced in 3T3-L1 mature adipocytes compared with 3T3-L1 pre-adipocytes, while the expression of these proteins was abrogated by 100 µM of FA.

In contrast, we found that HO-1 was significantly decreased in 3T3-L1 mature adipocyte compared with 3T3-L1 pre-adipocytes, while FA increased the expression of HO-1 compared with the corresponding control. These results suggested that FA suppresses adipocyte differentiation through the activation of HO-1 during the adipogenesis of 3T3-L1 cells.

### 2.2. FA Increases the Expression of HO-1 in 3T3-L1 Treated with a HO-1 Antagonist 

**Figure 2:**
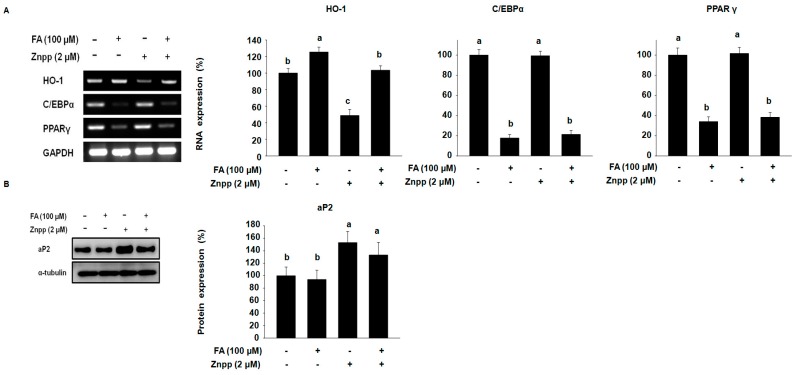
FA increases the expression of HO-1 in 3T3-L1 cells treated with a HO-1 antagonist. (**A**) Cells were harvested after 5 days. Their extracted total RNA was subjected to RT-PCR to analyze the level of HO-1, C/EBPα, and PPARγ. The mRNA expression level was normalized against GAPDH; (**B**) 3T3-L1 cells differentiated with MDI (3-isobutyl-1-methylxanthine, dexamethasone, and insulin) in the presence or absence of 2 μM ZnPP and 100 μM FA. Cells harvested after 5 days were subjected to Western blots to analyze the level of aP2. The protein expression level was normalized against α-tubulin. The experiments were performed in triplicate. Results were analyzed by one-way ANOVA and Duncan’s multiple range test. The *p*-value in the multiple comparison results (e.g., a, b, c, d, and e) indicate significant differences among the groups (*p* < 0.05).

To examine whether the suppression of adipocyte differentiation by FA is due to the increased expression of HO-1, 3T3-L1 cells were cultured in MDI with FA and/or a HO-1 antagonist (zinc protoporphyrin, ZnPP) for 8 days. As shown in Figure 2A, RT-PCR revealed that FA increased the expression of HO-1 but decreased the expression of C/EBPα and PPARγ compared with the corresponding control. In contrast, ZnPP inhibited the expression of HO-1 and slightly activated the expression of C/EBPα and PPARγ. Interestingly, FA treatment enhanced the expression of HO-1 and dramatically suppressed both C/EBPα and PPARγ in 3T3-L1 cells treated with ZnPP. 

Consistent with these observations, FA decreased the expression of aP2, while ZnPP enhanced the expression of aP2, as shown in Figure 2B. The combination treatment of FA suppressed ZnPP-induced aP2 expression in 3T3-L1 cells, indicating FA may be a negative regulator of adipocyte differentiation through the activation of HO-1. 

### 2.3. FA Attenuates the Expression of Fatty Acid Synthases and Intracellular Lipid Accumulation in 3T3-L1 Treated with the Presence of ZnPP

**Figure 3:**
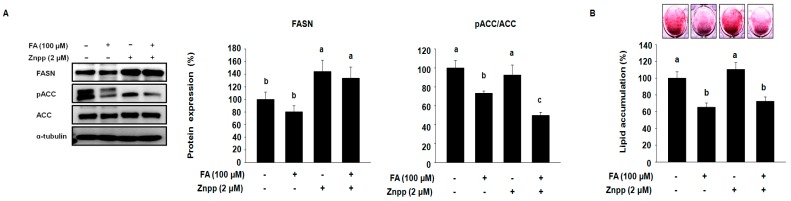
FA attenuates the expression of fatty acid synthases and intracellular lipid accumulation in 3T3-L1 cells treated with the presence of ZnPP. (**A**) Cell lysates differentiated by MDI in the presence or absence of 2 μM ZnPP and 100 μM FA for 5 days were subjected to Western blot analysis to probe the levels of FASN, pACC, and ACC. The protein expression level was normalized against ACC and α-tubulin. The experiments were performed in hexa-plicate; (**B**) Lipid accumulations were measured by an ORO assay in 3T3-L1 cells differentiated in the presence or absence of 2 μM ZnPP and 100 μM FA for 8 days, and the absorbance was determined at 490 nm. The results were analyzed using a one-way ANOVA and Duncan′s multiple range test. The experiments were performed in triplicate. The *p*-value in the multiple comparison results (e.g., a, b, c, d, and e) indicated significant differences among the groups (*p* < 0.05).

During the progression of adipogenesis, intracellular lipid synthesis occurs by the activation of fatty acid synthesis enzymes such as FASN and ACC. Western blots were conducted to further investigate whether FA attenuates pre-adipocytes differentiation into mature adipocytes and intracellular lipid synthesis in 3T3-L1 cells. As shown in Figure 3A, the expression of FASN was slightly decreased in 3T3-L1 cells treated with FA compared to the corresponding control, while ZnPP stimulated the expression of FASN. Moreover, we observed that FA repressed the protein levels of FANS in 3T3-L1 cells treated with ZnPP. The phosphorylation of ACC was significantly decreased by FA in 3T3-L1 cells treated with ZnPP. 

To confirm whether FA suppresses intracellular lipid accumulation in 3T3-L1 cells treated with ZnPP, ORO staining was performed. Lipid accumulation decreased with a similar tendency in FA-treated 3T3-L1 cells with and without ZnPP, indicating that FA has sufficient functional activity to induce HO-1 protein, even if 3T3-L1 cells were exposed to an HO-1 antagonist (Figure 3B). Moreover, FA may inhibit the progression of intracellular lipid accumulation in 3T3-L1 cells.

### 2.4. Effects of FA on Early Stage Adipocyte Differentiation Factors by Activating HO-1 in 3T3-L1 Cells

**Figure 4:**
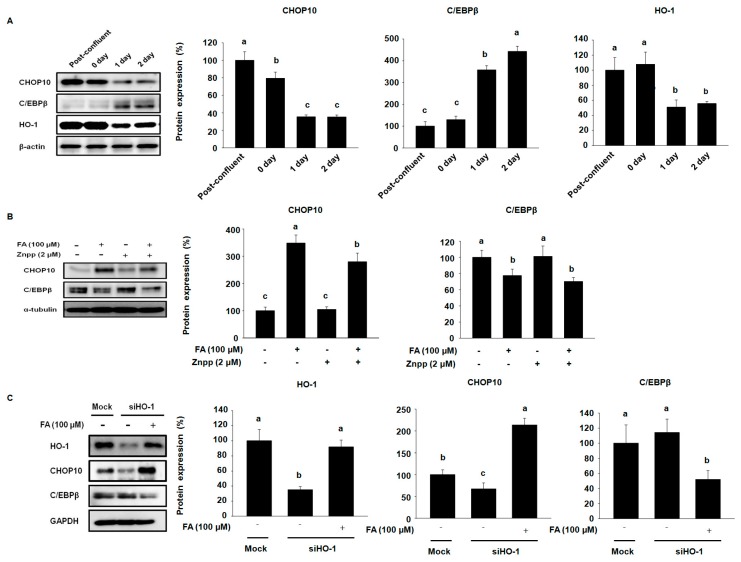
Effects of FA on early stage adipocyte differentiation factors by activating HO-1 in 3T3-L1 cells. (**A**) Cells were differentiated with 3-isobutyl-1-methylxanthine (IBMX), dexamethasone, and insulin. Proteins were harvested at the indicated time points and subjected to Western blot analysis to probe the levels of CHOP10, C/EBPβ, and HO-1. The protein expression level was normalized against β-actin; (**B**) 3T3-L1 cells were differentiated by MDI in the presence or absence of 2 μM ZnPP and 100 μM FA for 24 h and subjected to Western blot analysis to assess the levels of CHOP10 and C/EBPβ. The protein expression level was normalized against α-tubulin; (**C**) Pre-adipocytes were transiently transfected with HO-1 siRNA (siHO-1) or control siRNA (Mock siRNA). Transfected 3T3-L1 pre-adipocyte cells were differentiated with MDI in the presence or absence of 100 μM FA for 24 h. The protein expressions were evaluated by Western blots to analyze the levels of HO-1, CHOP10, and C/EBPβ. The protein expression level was normalized against GAPDH. Results were analyzed by one-way ANOVA and Duncan′s multiple range test. The experiments were performed in triplicate. The *p*-value in the multiple comparison results (e.g., a, b, c, d, and e) indicate significant differences among the groups (*p* < 0.05).

Our results prompted us to hypothesize that FA-induced HO-1 decreases early stage adipocyte differentiation markers such as CHOP10 and C/EBPβ, and subsequently disrupts the progression of adipocyte terminal differentiation in 3T3-L1 cells. To confirm our hypothesis, we examined the protein expression of CHOP10, C/EBPβ, and HO-1 from post-confluency until 2 days after MDI treatment in 3T3-L1 cells. As shown in Figure 4A, Western blots showed that the expression of CHOP10 was down-regulated in 3T3-L1 cells treated with MDI at 1 day, while the expression of C/EBPβ was initiated to increase the protein expression of CHOP10. Interestingly, we observed that the expression of HO-1 showed a similar tendency to that of CHOP10. 

To evaluate whether CHOP10 and C/EBPβ were regulated by FA-induced HO-1, 3T3-L1 cells were differentiated in MDI with FA and/or ZnPP for 2 days. FA stimulated the expression of CHOP10 and decreased the expression of C/EBPβ compared with the corresponding control, while ZnPP showed an opposite trend on CHOP10 and C/EBPβ expression compared with FA-treated 3T3-L1 cells (Figure 4B). These data indicate that FA-induced HO-1 may regulate the expression of CHOP10 in the early stages of adipogenesis. 

We next employed siRNA to clarify whether HO-1 modulates CHOP10 and its downstream protein C/EBPβ by knocking down the expression of HO-1. The siHO-1 dramatically inhibited the expression of HO-1 and CHOP10, while siHO-1 transfected 3T3-L1 cells treated with FA had markedly stimulated expression of HO-1 and CHOP10 compared with the control mock siRNA transfected 3T3-L1 cells (Figure 4C). Moreover, siHO-1 increased the expression of CEBPβ (the reverse expression pattern of HO-1 and CHOP10), while siHO-1 transfected 3T3-L1 cells treated with FA showed suppressed expression of CEBPβ.

These results confirmed that HO-1 acts as a positive regulator of CHOP10 and modulates its downstream target CEBPβ. In addition, FA is a potent inducer of HO-1 that may interfere with the progression of adipogenesis through the activation of CHOP10 in 3T3-L1 cells.

## 3. Discussion

Obesity can lead to lipid metabolism disorder, ischemic heart disease, insulin resistance, and type 2 diabetes. Adipocytes mainly compose the adipose tissue in the human body. Thus, the proper regulation of adipocytes can decrease the incidence of obesity itself and the above-mentioned obesity-associated diseases. Although pharmacological therapeutics have been developed for treatment of obesity and obesity-associated diseases in the last two decades, they often have side effects and limited benefits [21]. Therefore, it is necessary to demonstrate the effectiveness of a natural bioactive compound that can properly suppress adipocyte differentiation. 

FA is an antioxidant polyphenol and food additive [16]. FA is commonly found in fruits, vegetables, and seeds [22]. It has been reported that FA exhibits a variety of biomedical properties, such as anti-inflammation, anti-aging, and anti-apoptotic effects, because of its antioxidant activities. However, whether FA has a beneficial effect in modulating adipocyte differentiation in 3T3-L1 cells and its mechanism is unknown. 

Here, we provide evidence that the adipogenesis of 3T3-L1 cells was decreased by FA-mediated HO-1 and CHOP10 activation. CHOP10 serves as a gatekeeper for the inhibition of adipocyte differentiation. We showed that MDI cocktail stimulated the inverse modulation between CHOP10 and C/EBPβ during the mitotic clonal expansion (MCE) stage of adipogenesis. However, FA-mediated HO-1 activation stimulated the expression of CHOP10 and subsequently disturbed the induction of C/EBPβ. Furthermore, we knocked down HO-1 expression to elucidate the relationship between HO-1 and CHOP10 in 3T3-L1 cells. Then, cells were differentiated with MDI in the presence or absence of FA. This experiment revealed that CHOP10 is a downstream target of HO-1, suggesting that FA may not only induce the expression of the HO-1/CHOP10 axis, but also act as a negative regulator of adipocyte differentiation in 3T3-L1 pre-adipocytes. 

C/EBPα and PPARγ are positive regulators of adipocyte differentiation, and were increased in MDI cocktail-treated 3T3-L1 cells. However, we observed that C/EBPα and PPARγ were significantly down-regulated in MDI cocktail-treated 3T3-L1 cells with FA treatment. A previous report showed that HO-1 inhibition resulted in increased mesenchymal stem cell differentiation into adipocytes and increased PPARγ [23]. 

We did not observe significant changes in C/EBPα and PPARγ induced by ZnPP (HO-1 inhibitor) in 3T3-L1 cells, but aP2 significantly increased following ZnPP treatment. FA decreased the expression of aP2 in ZnPP-treated 3T3-L1 cells, suggesting that FA-mediated HO-1 expression may partially contribute to the escape from terminal differentiation of adipogenesis because it disrupts the initiation of MCE in 3T3-L1 cells. 

Additionally, lipogenesis typically occurs during adipocyte differentiation through the activation of the triglyceride synthesis pathway in 3T3-L1 cells [14]. To explain the reduction of adipogenesis in 3T3-L1 cells treated with FA, the effects of FA on triglyceride synthesis enzymes were assessed. We analyzed the expression of FASN and the phosphorylation of ACC, and found that FASN was slightly decreased by FA—in line with the huge change in the phosphorylation of ACC. FA attenuated the expression of FASN and the phosphorylation of ACC in ZnPP-treated 3T3-L1 cells. Moreover, ORO staining showed a similar tendency in lipogenesis-associated protein expression. Thus, our results suggest that FA might reduce adipogenesis, which contributes to the reduction in intracellular triglyceride accumulation. 

## 4. Materials and Methods 

### 4.1. Materials 

Ferulic acid (≥98% purity) was purchased from Santa Cruz Biotechnology (Santa Cruz, CA, USA). Zinc protoporphyrin (ZnPP) and 3-isobutyl-1-methylxanthine (IBMX) (≥98% purity) were purchased from Santa Cruz Biotechnology. 3T3-L1 cells were obtained from the American Type Culture Collection (American Type Culture Collection, Manassas, VA, USA). Dulbecco′s modified Eagle′s medium (DMEM), bovine calf serum (BCS), fetal bovine serum (FBS), insulin, penicillin-streptomycin (P/S), and trypsin-EDTA were purchased from Gibco (Gaithersburg, MD, USA). Dexamethasone (DEX) (≥97% purity), isopropanol, ORO, and crystal violet were obtained from Sigma. Formaldehyde was purchased from DAEJUNG (DAEJUNG chemical, Shiheung, Gyeonggi, South Korea). Thiazolyl blue tetrazolium bromide (MTT) was purchased from Alfa Aesar Chemical Inc. (Alfa Aesar Chemical, Ward Hill, MA, USA). Ethidium bromide was purchased from Thermo Fisher Scientific (Thermo Fisher Scientific, San Jose, CA, USA). The Maxime RT PreMix KIT was obtained from iNtRON Biotechnology (iNtRON Biotechnology, Seoul, Korea). TRIzol reagent was purchased from Invitrogen (Invitrogen, Carlsbad, CA, USA). Unless otherwise noted, all chemicals were obtained from Sigma (Sigma-Aldrich, St. Louis, MO, USA). 

Antibodies specific for HO-1, CHOP10, C/EBPβ, C/EBPα, DGAT1, FASN, GAPDH, and α-tubulin were obtained from Santa Cruz.

### 4.2. Cell Culture 

3T3-L1 cells were grown in DMEM containing 3.7 g/L sodium bicarbonate, 1% P/S, and 10% BCS at 37 °C in 5% CO_2_. Two-day post-confluent cells were differentiated with 10% FBS and MDI differentiation cocktail (MDI: 0.5 mM IBMX, 1.0 μM DEX, and 4 μg/mL insulin). The medium was replaced with DMEM containing 4 μg/mL insulin and 10% FBS for 2 additional days. The medium was then replaced with fresh DMEM containing 10% FBS every other day until the indicated time point (days 6 to 8), and 25, 50, and 100 μM FA was dissolved in DMSO. The final concentration of DMSO was 0.001% in all experiments.

### 4.3. Cytotoxicity Assay

Pre-adipocytes (1 × 10^4^ cells/well) were seeded in 96-well plates. Cells were treated with up to 200 μM FA for 24 h. The treated cells were stained with MTT for 3 h at 37 °C in 5% CO_2_. Then, MTT formazan was eliminated 100 μL of DMSO and determined by ELISA reader Powerwave HT at 570 nm (Bio Tek, Winooski, VT, USA)

3T3-L1 pre-adipocytes (1 × 10^4^ cells/well) were incubated with DMEM with 10% BCS media overnight in 96-well plates. Cells were treated with up to 200 μM FA for 24 h. Crystal violet reagent was added to the 96-well plate and incubated for 30 min at 37 °C. Then, the supernatant was gently eliminated, and 100 μL of DMSO was added to extract the intracellular crystal violet in the 3T3-L1 cells. The crystal violet product was measured by ELISA reader Powerwave HT at 570 nm (Bio Tek, Winooski, VT, USA). The experiments were performed in hexa-plicate.

### 4.4. Oil Red O Staining 

3T3-L1 cells with differentiation induced were washed with PBS and fixed with 10% formaldehyde at 4 °C for 1 h. After washing with 60% isopropanol, the fixed cells were stained with 0.5% ORO in 60:40 (*v*/*v*) ORO/H_2_O for 20 min at room temperature and washed four times. Lipid accumulation was quantified using 100% isopropanol, which was used to elute ORO dye, and the absorbance was determined by an ELISA reader Wallac Victor 140 (PerkinElmer, Boston, MA, USA) at 490 nm. The experiments were performed in hexa-plicate.

### 4.5. Transfection (siRNA)

Pre-adipocytes (5 × 10^5^ cells/well) were seeded in six-well plates. Cells were transfected for 2 days using a predesigned siRNA control (Cat. No. SN-1003) and siRNA against HO-1 (sense 5′-CAG AUC AGC ACU AGC UCA U-3′; antisense 5′-AUG AGC UAG UGC UGA UCU G-3′) from Bioneer and the TransIT-TKO Transfection Reagent. Transfected cells were differentiated with a MDI differentiation cocktail (MDI: 0.5 mM IBMX, 1.0 μM DEX, and 4 μg/mL insulin) for 24 h. The experiments were performed in triplicate. 

### 4.6. Isolation of RNA and RT-PCR 

Differentiated cells were harvested using the TRIzol reagent according to the manufacturer’s protocol. The total RNA was reverse transcribed to cDNA using a Maxime RT PreMix KIT for 60 min at 45 °C and then 5 min at 95 °C. The cDNA was amplified using the specific primers listed in Table 1 and an RT-PCR system. The reaction was denatured at 95 °C for 5 min, annealed at 55 °C for 30 s, and extended at 72 °C for 1 min. The PCR products were electrophoresed in 1.5% (*v*/*v*) agarose gels, stained with ethidium bromide, and photographed. The signal intensity was quantified with GENE FLASH (SYGENE). The experiments were performed in triplicate.

### 4.7. Western Blot Analysis

Cells were harvested in lysis buffer containing phosphatase inhibitor cocktails II and III. Protein extracts (25 μg) were separated via SDS-PAGE and transferred to a PVDF membrane. The membranes were blocked with 5% non-fat dried milk and immunoblotted with primary antibodies specific for the indicated proteins overnight. Secondary antibodies conjugated with horseradish peroxidase (1:5000) applied for 4 h. The bands were visualized by enhanced chemiluminescence, and the proteins were detected using LAS Image software (Fuji, New York, NY, USA). The experiments were performed in triplicate.

### 4.8. Statistical Analysis

Differences among multiple groups were determined using a one-way analysis of variance (ANOVA) followed by Duncan′s multiple range test using the SAS 9.0 software (SAS Institute, Cary, NC, USA). Values with different superscript letters are significantly different, and results were considered significant if *p* < 0.05.

## 5. Conclusions

The present study revealed the possibility that FA stimulates the expression of HO-1, which retains the expression of CHOP10 and might disrupt the complex downstream cascade of adipogenesis and lipogenesis of 3T3-L1 cells. Thus, we suggest that FA may act as a positive regulator of HO-1 and may be an effective natural bioactive compound to disrupt the progression of the MCE stage of adipogenesis.

## Figures and Tables

**Table 1:** List of primers used in this study for RT-PCR.

Name	Forward (5′–3′)	Reverse (5′–3′)
HO-1	CTGTGTAACCTCTGCTGTTCC	CCACACTACCTGAGTCTACC
PPARγ	CTGTGAGACCAACAGCCTGA	AATGCGAGTGGTCTTCCATC
C/EBPα	TGAAGGAACTTGAAGCACAA	TCAGAGCAAAACCAAAACAA
GAPDH	AACTTTGGCATTGTGGAAGG	ACACATTGGGGGTAGGA
